# Endocrine resistance in breast cancer: Current status and a perspective on the roles of miRNAs (Review)

**DOI:** 10.3892/ol.2013.1405

**Published:** 2013-06-17

**Authors:** JICHUN ZHOU, RONGYUE TENG, QINCHUAN WANG, CHENPU XU, JUFENG GUO, CHAO YUAN, JIANGUO SHEN, WENXIAN HU, LINBO WANG, SHUDUO XIE

**Affiliations:** Department of Surgical Oncology, Sir Run Run Shaw Hospital, Zhejiang University School of Medicine, Hangzhou, Zhejiang 310016, P.R. China

**Keywords:** miRNAs, breast cancer, endocrine resistance

## Abstract

Current endocrine therapies for females with estrogen receptor-positive breast cancer have facilitated substantial improvements in outcomes. The effectiveness of endocrine therapy is limited by either initial *de novo* resistance or acquired endocrine resistance. Multiple mechanisms responsible for endocrine resistance have been proposed, including deregulation of various components of the estrogen receptor (ER) pathway, alterations in cell cycle and cell survival signaling molecules, and the activation of escape pathways. Dysregulation of miRNA expression has been associated with experimental and clinical endocrine therapy resistance. miRNAs are pivotal to understanding the complex biological mechanism of endocrine resistance, and may serve as novel candidate predictive and prognostic surrogates and therapeutic targets. This review focuses on current progress concerning the roles of miRNAs in endocrine resistance, and discusses the challenges and opportunities for implementing miRNA-based assays and treatment for patients with endocrine-resistant breast cancer.

## Contents

1. Introduction
2. Brief overview of the mechanisms of endocrine resistance in breast cancer
3. miRNA: Biogenesis and function
4. miRNAs and breast cancer
5. Role of miRNAs in the endocrine resistance of breast cancer
6. Future prospects
7. Conclusion

## 1. Introduction

Although extensive research into the molecular mechanisms involved in breast cancer has been performed, challenges remain in the early diagnosis and management of patients with breast cancer, such as unpredictable responses and the development of resistance to adjuvant therapies (including endocrine therapy). Current endocrine therapies for females with estrogen receptor (ER)-positive breast cancer have led to substantial improvements in outcomes (1). ER-targeted therapy has improved the quality of life and survival of millions of females with breast cancer worldwide in the past three decades, but its success has been limited by *de novo* and acquired resistance. Significant efforts have been undertaken to resolve the molecular mechanisms that lead to endocrine resistance. miRNAs are evolutionarily conserved short non-coding RNA genes that were first identified over a decade ago (2–4). According to various studies over the past decade, miRNAs are an important and prevalent regulatory layer of gene expression that acts at the post-transcriptional level (5,6), and have also been implicated to be pivotal in common human diseases, including cancer (7). miRNAs are rapidly emerging as a novel class of biomarkers with a unique set of biological and chemical properties that makes them extremely attractive candidates for clinical implementation in cancer. Since miRNA deregulation in breast cancer was first reported in 2005 (8), there have been numerous studies on the aberrant expression of various miRNAs and their roles in the mechanisms of endocrine resistance in breast cancer (9,10). miRNAs may have a significant role in the development of endocrine resistance, as well as affecting the progression and proliferation of breast cancer cells. miRNA-based analysis is an area of research that has rapidly accelerated since it began. This is due to the significant effect of miRNA-mediated gene regulation and the clear potential of these extremely small molecules for future diagnostic, predictive and therapeutic applications. miRNAs may significantly enhance the under-standing of the mechanisms of endocrine resistance, and provide real-time information concerning the development of such resistance.

This review addresses efforts to understand how the miRNA profile is altered upon the development of resistance; the critical regulatory role of miRNAs in conferring resistance to commonly used endocrine agents; and how these differentially expressed miRNAs may serve as prognostic and predictive markers and novel therapeutic targets for overcoming endocrine resistance.

## 2. Brief overview of the mechanisms of endocrine resistance in breast cancer

The term endocrine therapy is applied to breast cancer treatments that target the ER by blocking receptor binding with an antagonist or by depriving the tumor of estrogen. The ER, which has nuclear (genomic) and non-nuclear (non-genomic) functions, is the predominant driver of the majority of types of breast cancer (11). With at least 70% of breast cancers exhibiting high ER expression, which is known to contribute to tumor growth and progression (12), Beatson’s findings revolutionized the management of breast cancer, leading to the discovery of the selective ER modulator (SERM), tamoxifen. This agent has been the mainstay endocrine therapy for breast cancer for the past 25 years (13). Tamoxifen was shown to improve survival in early breast cancer (1) as well as quality of life for patients with advanced breast cancer (14). Over the past decade, several novel endocrine agents against ER-positive breast cancer, which either lower the estrogen ligand for the ER (aromatase inhibitors) or degrade the ER (ICI 182,780 fulvestrant), have also demonstrated activity in various clinical settings (15,16). These agents, which result in a more effective inhibition of ER signaling, have been demonstrated to be clinically effective and are now an indispensable part of the present treatment strategies for breast cancer (17–22).

Regardless of these significant advances in the treatment of patients with initially hormone-sensitive breast cancer, resistance to all forms of endocrine therapy remains a major clinical issue; a significant proportion of patients experience recurrence caused by intrinsic or acquired resistance to endocrine agents within 15 years (20). At present, resistance to endocrine therapy is considered to be a progressive, step-wise phenomenon induced by the selective pressure of hormonal agents. Breast cancer cells are converted from an estrogen-dependent phenotype, which is responsive to endocrine manipulation, to a non-responsive phenotype and eventually to an estrogen-independent phenotype. The molecular mechanism of endocrine resistance involves alterations to the ER and its co-regulators, receptor tyrosine kinase (RTK) signaling, cell cycle regulators, the cell survival pathway and apoptosis (23,24). The mechanism of endocrine resistance is further complicated by the results of a study that utilized a next generation sequencing (NGS) approach and a novel bioinformatics model to compare the transcriptomes of tamoxifen-sensitive and -resistant breast cancer cells (25). The authors identified differential expression of 1,215 mRNA and 513 small RNA transcripts clustered around ERα function, cell cycle regulation, transcription/translation and mitochondrial dysfunction. The extent of the alterations observed at multiple levels of gene regulation demonstrates the ability of the tamoxifen-resistant cells to modulate global gene expression (25).

Although knowledge of the mechanism of endocrine resistance is developing, a more detailed understanding of the molecular mechanisms and regulatory pathways involved in breast cancer cells is required to improve the design of novel anti-tumor drugs and inform the selection of optimal therapeutic strategies.

## 3. miRNA: Biogenesis and function

miRNAs are a contemporary class of tiny non-coding endogenous RNA molecules that are only 18–25 nucleotides in length. It has been proposed that the discovery of miRNAs as regulators of gene expression represents a paradigm-changing event in biology and medicine (26). miRNA was first described in *Caenorhabditis elegans* in the laboratory studies of Victor Ambros and Gary Ruvkun (27,28). The authors identified lin-4, which was originally considered to be a biological entity specific to the *C. elegans* nematode (27,28). Subsequently, miRNA research has rapidly expanded, with the realization that miRNAs are critical to the development of multicellular organisms and the basic functions of cells (29). At present, ∼1,100 miRNAs and 16,228,619 predicted miRNA target sites in 34,911 distinct 3′UTR from isoforms of 19,898 human genes have been identified in the human genome (http://www.microrna.org/) (30). As they are fundamental to genetic regulation, miRNAs are estimated to regulate approximately one-third of all human transcripts (31).

It is well-recognized that miRNAs are involved in the regulation of multiple cellular processes, including proliferation, apoptosis, cell-cycle regulation and differentiation (29). Aberrant expression and function of miRNAs have been associated with numerous diseases and disorders (29). Notably, abnormalities in miRNA activity have been implicated in the initiation and progression of cancer, as miRNA alterations are ubiquitous among human cancers, where they may function as oncogenes or tumor suppressor genes. miRNAs function as key gene regulators capable of silencing gene expression at the post-transcriptional level.

## 4. miRNAs and breast cancer

In the last 5 years, miRNA studies of breast cancer have represented a valuable area of research, having resulted in new knowledge on the molecular basis of the disease, tools for molecular classification, new markers with diagnostic and prognostic relevance, as well as the identification of novel breast cancer-predisposing genes (32). It has been suggested that, as well as altering estrogen-responsive gene expression, miRNAs may simultaneously achieve a secondary level of post-transcriptional regulation of pathways implicated in breast cancer and resistance to endocrine therapy (33).

The association between miRNAs and breast cancer has been elucidated by studies investigating the expression of miRNAs in breast cancer cell lines and clinical samples. miRNA expression studies in breast cancer have indicated the importance and potential use of miRNAs as disease classifiers and prognostic tools. Lu *et al* analyzed the systematic expression of 217 miRNAs from 334 samples (including breast cancer) and observed that the miRNA profiles were informative, reflecting the developmental lineage and differentiation state of the tumors, with a general downregulation of miRNAs noted in the tumors compared with normal tissues (34). Another study identified 29 miRNAs that were differentially expressed in breast cancer tissue compared with normal tissue, as well as a further set of 15 miRNAs that were able to correctly discriminate between tumor and normal tissue (8). Increasing data has revealed that miRNAs modulate the network of signal transduction pathways associated with drug resistance by upregulating drug efflux transporters and anti-apoptotic proteins, undergoing epithelial-mesenchymal transition (EMT) and forming cancer stem cells [review (35)]. The involvement of miRNA-10b (36), -335 (37), -373 and -520c (38) has been identified in the development of breast cancer metastases [review (39)].

Furthermore, present findings have indicated that miRNAs are involved in the majority of stages of breast cancer development, such as regulating self-renewal, the tumorigenicity of breast cancer cells (40,41), apoptosis and angiogenesis [review (42)].

## 5. Role of miRNAs in the endocrine resistance of breast cancer

It is considered that miRNAs are associated with the ER pathway and other pathways involved in endocrine resistance (9,43). Although their involvement is apparent, the study of the specific pathways they affect and the mechanisms they aid in regulating has only recently begun. The incorporation of miRNA regulation into the current models of the molecular mechanism of endocrine resistance in breast cancer is likely to be essential to achieve a complete understanding of endocrine resistance.

### miRNA expression is closely correlated with endocrine resistance

#### miRNAs and the ER

The ER remains the best predictor for endocrine therapy. However, it is reasonable to propose that miRNAs may function as endocrine resistance stimulators and inhibitors, and be involved in associated pathways by directly or indirectly regulating genes. ERα mRNA has a long 3′-untranslated region (3′UTR) of ∼4.3 kb, which has been reported to reduce mRNA stability and comprise evolutionarily conserved miRNA target sites, enabling it to be regulated by miRNAs.

The first indication of the post-transcriptional regulation of ERα by miRNA in the context of breast cancer was observed by Adams *et al* (43). Two miRNA-206-targeting sites were identified *in silico* within the 3′UTR of human ERα mRNA, and transfection of MCF-7 cells with pre-miRNA-206 specifically decreased the ERα mRNA levels. Notably, the miRNA-206 levels were higher in ERα-negative MB-MDA-231 cells compared with ERα-positive MCF-7 cells. miRNA-206 expression was markedly inhibited by ERα agonists, although not by ERβ agonists or progesterone. The authors suggested that miRNA-206 may function in a mutually negative feedback loop to temporally regulate ERα expression (43). Another study also demonstrated that miRNA-206 is inversely correlated with ERα expression (44). Furthermore, transfecting MCF-7 cells with an expression plasmid for pre-miRNA-206 reduced ERα mRNA expression and the basal expression levels of the progesterone receptor (PR), cyclin D1 and pS2 (well-established ERα-regulated genes), and inhibited cell proliferation in a 17β-estradiol (E2)-independent manner. The authors suggested that miRNA-206 may be a novel candidate for endocrine therapy that specifically targets ERα in breast cancer (44). By contrast, an additional two studies did not identify E2-independent miRNA-206 regulation of expression, which the authors surmised may be consistent with the apparent lack of miRNA-206 expression in MCF-7 cells (45,46).

Pandey and Picard suggested that miRNA-22 represses ERα expression most markedly and directly through the 3′UTR, resulting in a reduction in estrogen signaling (46). In addition, Xiong *et al* also revealed that there was a significant inverse association between the miRNA-22 levels and ERα protein expression in five breast cancer cell lines and 23 clinical biopsies (47). miRNA-22 was frequently down-regulated in the ERα-positive human breast cancer cell lines and clinical samples. The authors suggested that miRNA-22 may be pivotal in the pathogenesis of breast cancer by direct involvement in the regulation of ERα (47). miRNA-221/222 was also revealed to be involved in the suppression of ERα protein expression, which is detailed in the following section (48,49). In addition, Lau *et al* examined the expression of miRNA-449a/b in 60 fresh-frozen breast tumor samples, and observed that the expression of miRNA-449a/b was inversely correlated with tumor grade and markedly associated with the estrogen receptor status of the tumor. The authors suggested that miRNA-449a/b may affect tamoxifen sensitivity in breast cancer cells (50).

Maillot *et al* demonstrated that a set of 23 miRNAs (including miRNA-181a, -21, -181b, -26a, -200c, -26b, -27b and -23b) were downregulated by E2 in various ER-positive breast tumor cell lines (45). The RNA precursors of the miRNAs were the primary transcriptionally repressed targets of ER. In addition, transcriptome analysis revealed that the E2-repression of miRNA-26a and -181a regulated numerous genes associated with cell growth and proliferation, including the progesterone receptor (PGR) gene (45).

miRNAs are also able to affect estrogen-regulated gene expression by inhibiting the expression of the coactivator SRC-3/AIB1/NCOA3. miRNA-17-5p was demonstrated to inhibit the translation of SRC-3/AIB1/NCOA3 in a study by Hossain *et al* (51). Transfection of CHO-K1 cells with ERα and miRNA-17-5p inhibited E2-stimulated ERE-driven luciferase reporter activity by 50%. This study also demonstrated that the transfection of MCF-7 cells with miRNA-17-5p reduced E2-induced proliferation and endogenous cyclin D1 transcription (51). Furthermore, the expression level of Argonaute-2 (Ago2), the catalytic subunit of the RNA-induced silencing complex (RISC) that mediates miRNA-dependent cleavage/degradation, is higher in ERα-negative/human epidermal growth factor receptor 2 (HER2)-positive cells compared with ERα-positive/HER2 negative human breast cancer cell lines and tumors (52). E2 and the ERα-agonist, propyl pyrazole triol (PPT), were able to increase the expression of Ago2 protein in MCF-7 cells. In addition, the high expression of Ago2 in ERα-negative cells was severely limited by the inhibition of the epidermal growth factor receptor (EGFR)/mitogen-activated protein kinase (MAPK) signaling pathway, indicating that EGF acts through the MAPK pathway to increase Ago2 protein stability. However, the mechanism by which E2 and PPT increase Ago2 protein levels, potentially through ERα, remains poorly understood. Ago2 overexpression in MCF-7 cells increased the ERα protein levels by three-fold, regardless of also increasing the levels of miRNA-206 that suppress ERα (52). This study indicated that other factors involved in miRNA biogenesis may also be involved in the mutual regulation between ER and miRNAs.

#### miRNAs and other endocrine resistance associated pathways

In addition to estrogen-mediated gene expression, hormone-regulated miRNAs may provide an additional level of post-transcriptional regulation for signaling pathways critically involved in the endocrine resistance of breast cancer. The expression levels of a number of miRNAs in ER-positive breast cancer have been directly associated with responses to endocrine therapy (9).

**Figure 1.**
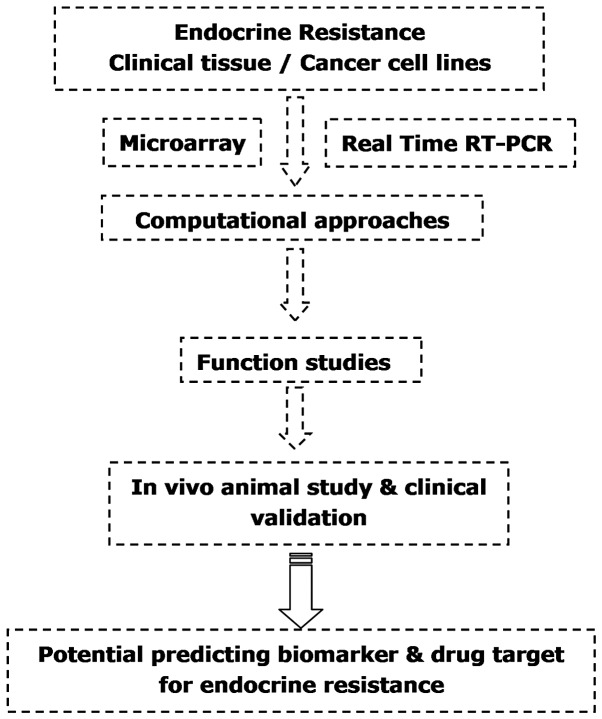
Typical model of investigation on the role of miRNAs in endocrine resistance in breast cancer.

The model of research (Fig. 1) in the majority of studies investigating the potential roles of aberrantly expressed miRNAs in acquired endocrine resistance is as follows: i) microarray data are utilized to compare endocrine-resistant and -sensitive breast cancer cells and clinical samples, revealing the differential expression of miRNAs; ii) computational approaches (shown in Table I) are utilized to predict the potential targets and signaling pathways of the aberrantly expressed miRNAs *in silico*; iii) functional studies *in vitro* are implemented to validate the predicted regulatory roles of the miRNAs via ectopic expression and downregulation; and iv) to support the significance of the studies performed in cell lines, clinical data are analyzed and *in vivo* animal studies are conducted to further confirm the association between miRNAs and endocrine resistance.

Miller *et al* investigated the role of miRNAs in acquiring resistance to tamoxifen by comparing the miRNA profiles of tamoxifen-resistant with tamoxifen-sensitive MCF-7 breast cancer cell lines by microarray analysis (9). The results showed the significant upregulation of eight miRNAs and marked downregulation of seven miRNAs in tamoxifen-resistant MCF-7 breast cancer cells compared with tamoxifen-sensitive cells. Furthermore, the expression levels of miRNA-221 and -222 were also significantly elevated in HER2/neu-positive primary human breast cancer tissues, which are known to be resistant to endocrine therapy, compared with HER2/neu-negative tissue samples. The ectopic expression of miRNA-221/222 rendered the parental MCF-7 cells resistant to tamoxifen. The protein level of the cyclin-dependent kinase inhibitor 1B (p27Kip1) cell cycle inhibitor, a known target of miRNA-221/222, was reduced by 50% in tamoxifen-resistant cells and 28–50% in tamoxifen-resistant cells that overexpressed miRNA-221/222 (9). This was the first study to demonstrate a correlation between miRNA-221/222 expression and HER2/neu overexpression in primary breast tumors that are generally resistant to tamoxifen therapy.

Almost simultaneously, another study revealed the distinct expression of a panel of miRNAs in a comparison between ERα-positive and -negative breast cancer cell lines and primary tumors, using a custom miRNA microarray platform containing 515 miRNAs (48). Similar to the previously mentioned study, the miRNA-221/222 levels were also observed to be higher in ERα-negative breast cancer cell lines and human breast tumors compared with those that were ERα-positive. Furthermore, two miRNA-221 and -222 seed elements were identified in the 3′UTR of ERα. Ectopic expression of miRNA-221 and -222 in ERα-positive MCF-7 and T47D cells suppressed the expression of ERα protein, but not mRNA. Notably, this rendered the cells resistant to tamoxifen. By contrast, knockdown of miRNA-221 and -222 in ERα-negative MDA-MB-468 cells partially restored the ERα protein expression and increased tamoxifen-induced apoptosis. These findings indicated that miRNA-221 and -222 are pivotal in the regulation of ERα expression, and may serve as potential targets for restoring ERα expression and responding to endocrine therapy in a subset of breast cancers (48). Another study revealed a role of miRNA-221/222 in the underlying mechanism of acquired fulvestrant resistance in MCF7 cells (53). The overexpression of miRNA-221/222 in ERα-positive cell lines confers hormone-independent growth and fulvestrant resistance through offsetting the effects of E2 depletion or fulvestrant-induced cell death. Furthermore, potential miRNA-221/222 targets were identified by global gene expression profiles, revealing that miRNA-221/222 overexpression resulted in the deregulation of multiple oncogenic signaling pathways associated with drug resistance (53). β-catenin is activated by miRNA-221/222, while transforming growth factor β (TGF-β)-mediated growth inhibition is repressed. These effects are critical for estrogen-independent growth and fulvestrant resistance (53). Lau *et al* studied the expression profiles of >600 miRNAs in a pair of tamoxifen-sensitive ZR75 and -resistant AK47 breast cancer cell lines (50). A total of 65 miRNAs were identified as being significantly downregulated in the tamoxifen-resistant cell line, while 44 miRNAs were revealed to be upregulated. Consistent with the findings discussed previously, the profiling results also indicated that miRNA-221 and -222 were overexpressed in the tamoxifen-resistant breast cancer cells (50).

**Figure 2.**
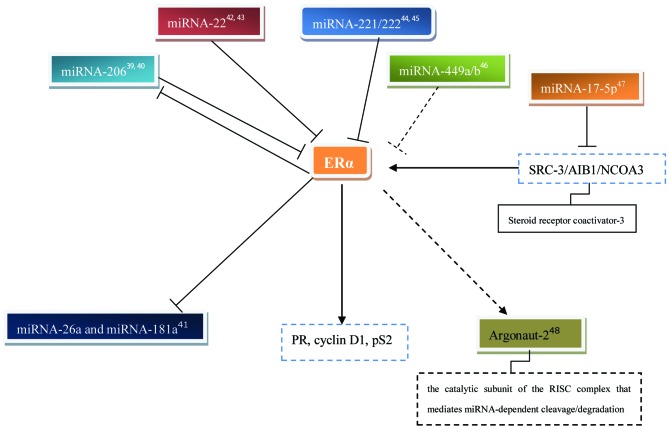
An overview of the interaction between ER and miRNAs. ER, estrogen receptor.

Manavalan *et al* used microarray to identify 97 differentially expressed miRNAs that were regulated by 4-hydroxytamoxifen in a comparison between MCF-7 endocrine-sensitive and -resistant breast cancer cells (49). The microarray expression data showed that the expression of miR-10a, -22, -29a, -125b, -181a and-222 was lower in EtOH-treated MCF-7 cells compared with that in LY2 cells. In contrast, the expression of miR-21, -93 and -200a,b, and c was lower in EtOH-treated LY2 cells compared with in MCF-7 cells. Of these miRNAs, only miR-21 and miR-181a were regulated by E2 in MCF-7 cells. The authors also observed that miRNA-221/222 was overexpressed in TAM-resistant breast cancer cell lines, suppressing ERα expression. Bioinformatic analysis to evaluate the biological significance of miRNAs identified 36 potential gene targets among those regulated by 4-hydroxytamoxifen in MCF-7 cells. The targets of the miRNAs were predicted *in silico* using identification software. Functional and network analyses of the changes in differential miRNA gene expression were performed using Ingenuity Pathways Analysis (IPA) 8.8 (Ingenuity^®^ Systems, Redwood City, CA, USA; http://www.ingenuity.com). Agreement in the direction of anticipated regulation was detected for 12 putative targets. Pyruvate decarboxylase regulator (*PDCD4)*, B-cell CLL/lymphoma 2 (*BCL2)*, cytochrome P450 1B1 (*CYP1B1)* and v-erb-b2 erythroblastic leukemia viral oncogene homolog 3 (*ERBB3)* were revealed to be differentially expressed between the two cell lines. However, the role of the interaction between the target genes and miRNAs for endocrine resistance (Fig. 2) were not clearly confirmed. The miRNAs with opposite expression patterns between the two cell lines may have been involved in the endocrine resistance (49).

To investigate the role of miRNAs in resistance to fulvestrant, a study used experimental microarray data to compare the global miRNA expression patterns between fulvestrant-resistant MCF7-FR cells and their drug-sensitive parental ER-positive MCF7 cells (10). The authors identified 14 downregulated miRNAs (let-7i, miRNA-181a, -191, -199b, -204, -211, -212, -216, -328, -373, -424, -204 and -191) in MCF7-FR cells, and then used TargetScan and the Parallel Implicit Time-Integration Algorithm (PITA) to predict potential target genes. Consequently, pathway analyses predicted that these miRNAs would regulate well-described cancer-associated signaling pathways, such as the TGF-β, Wnt, MAPK and mammalian target of rapamycin (mTOR) pathways (10). It has been reported that fulvestrant-resistant breast cancer cells were able to utilize Wnt/β-catenin and EGFR/ErbB2 signaling pathways to establish estrogen-independence and autocrine-regulated proliferation (54). These results suggest a significant role for miRNA-regulated gene expression in the onset of breast cancer anti-estrogen resistance. An enhanced understanding of this phenomenon may lead to improved therapies for this often fatal condition.

miRNA-342 was observed to be significantly downregulated in breast tumor samples and cell models of tamoxifen resistance. Restoring miRNA-342 expression in the resistant MCF-7 cell lines resensitized these cells to tamoxifen-induced apoptosis. In addition to a role in apoptosis, IPA revealed that miRNA-342 may affect multiple stages of the cell cycle through cyclin B1 suppression, numerous breast cancer 1 (BRCA1) activities, p53 cell cycle checkpoint function and phosphatase and tensin homolog (PTEN) tumor suppressor activity. Overall, these results suggest that miRNA-342 modulates the expression of genes involved in tamoxifen-mediated tumor cell apoptosis and cell cycle progression (55).

Lu *et al* observed the significant overexpression of miRNA-181b, -221 and -222 in tamoxifen-resistant MCF-7 cells. In addition, anti-miRNA-222 or -181b, in combination with tamoxifen, suppressed the growth of tamoxifen-resistant xenografts in mice through tissue inhibitor of metalloproteinase 3 (TIMP3), which is a common target of miRNA-221, -222 and -181b. Downregulation of TIMP3 enabled the growth of tamoxifen-resistant cells by alleviating its inhibitory effect on a disintegrin and metallopeptidase domain 10 (ADAM10) and ADAM17, which are essential for tamoxifen-resistant cell growth. Furthermore, the authors indicated that TIMP3 and the miRNAs may modulate EGF-induced MAPK and AKT phosphorylation. Based on these findings, the authors proposed that miRNA-221, -222 and -181b facilitate growth factor signaling in tamoxifen-resistant breast cancer by down-regulating TIMP3, and corresponding anti-miRNAs may be utilized to render these tumors responsive to tamoxifen (56).

The absence of clinical cross-resistance among the three aromatase inhibitors (AIs) and tamoxifen indicates that the mechanisms of resistance to these endocrine therapy agents are not identical (57). Masri *et al* identified 49 hormone-responsive miRNAs in hormone-refractory cell lines using microarray analysis. A number of hormone-responsive miRNAs were inversely expressed between the androgen independent (AI), long-term estrogen-deprived (LTEDaro) and tamoxifen-resistant cell lines. Subsequently, the authors focused on miRNA-128a, which was hormone-responsive and differentially overexpressed in letrozole-resistant cell lines. Furthermore, TGF-β receptor 1 (TGFβR1) and SMAD2 were confirmed to be targets of miRNA-128a. The inhibition of endogenous miRNA-128a resulted in resensitization of the letrozole-resistant lines to the growth inhibitory effects of TGF-β (33).

HER2Δ16 (a clinically important oncogenic isoform of HER2) expression promotes endocrine resistance in HER2/ERα-positive breast tumors via the suppression of miRNA-15a/16, which targets BCL-2 (58). The authors suggested that their study provided a template for unique therapeutic interventions, which combine tamoxifen with the modulation of miRNAs (58). Furthermore, this study provided unique insights into the molecular complexity of endocrine-resistant Her2- and ERα-positive breast cancer. It is notable that this study demonstrated a new approach to investigating the role of miRNAs in endocrine resistance, which utilized bioinformatics tools to select endocrine resistance-associated protein-targeting miRNAs.

The 14-3-3 family member and conserved protein, 14-3-3 ζ, is upregulated by tamoxifen, and this increased expression is correlated with early disease recurrence (59). A study revealed that the tamoxifen upregulation of 14-3-3 ζ results from its ability to rapidly downregulate miRNA-451 that specifically targets 14-3-3 ζ (6). Increasing the level of miRNA-451, by overexpression, downregulated 14-3-3 ζ. This, in turn, suppressed cell proliferation and colony formation, markedly reduced the activation of HER2, EGFR and MAPK signaling, increased apoptosis and, notably, restored the growth-inhibitory effectiveness of SERMs in endocrine-resistant cells (60).

In addition to the role of miRNAs in acquired endocrine resistance, a study investigated their critical roles in estrogen-independent growth, which results in intrinsic tamoxifen resistance (61). An *in vivo* selection system was used against an miRNA library. ER-positive MCF-7 cells were initially infected with the pooled library and subsequently, the cells were orthotopically injected into female nude mice depleted of estrogen. Further studies highlighted the enrichment of miRNA-101, which alone is able to promote estrogen-independent growth and lead to tamoxifen resistance without targeting the ER. Finally, the direct suppression of membrane associated guanylate kinase (Magi-2) by miRNA-101 was demonstrated to provide a molecular link for miRNA-101-mediated activation of Akt through the suppression of PTEN activity. Given these findings, the authors suggested that miRNA-101 may serve as a biomarker for the subpopulation of ER-positive patients with breast cancer that are intrinsically resistant to tamoxifen (61).

#### miRNAs may predict responses to endocrine therapy

The ability of miRNAs to be used as predictive biomarkers has been demonstrated in numerous studies (62,63). miRNAs have also been shown to exhibit potential for predicting responses to endocrine therapy in breast cancer.

miRNA profiling was used by Rothé *et al* as a complementary tool to assess whether miRNA expression was able to predict the clinical outcome of patients with breast cancer who were treated with tamoxifen alone (64). The authors evaluated the expression of miRNA-210 in a cohort of 89 ER-positive patients with breast cancer. A high level of miRNA-210 expression was observed to be associated with a higher risk of recurrence compared with lower levels of miRNA-210. Therefore, miRNA-210 was shown to be associated with a poor clinical outcome in tamoxifen treatment (64).

A study investigated the association between five putative candidate miRNAs and outcome in ERα-positive patients with breast cancer who received tamoxifen therapy for the advanced disease (65). The authors revealed an association between high expression levels of miRNA-30a-3p, -30c and -182 and clinical benefit/longer progression-free survival (PFS). Subsequently, the authors searched the published predicted target databases and none of these miRNAs were identified to have ER mRNA as a target, suggesting that the effects of the studied miRNAs on responses to tamoxifen were indirectly associated with the modulation of ER levels. Furthermore, the authors utilized global testing pathway analysis together with gene expression data to clarify the underlying mechanisms by which these miRNAs affected the outcome of tamoxifen treatment. miRNA-30c was shown to be positively associated with ERα and negatively associated with EGFR. Furthermore, miRNA-30c and -30a-3p were negatively correlated with the ras-related C3 botulinum toxin substrate 1 (RAC1) signaling pathway, which is driven by platelet-derived growth factor receptor α (PDGFR-α). Pathway analysis also showed that miRNA-30a-3p was positively correlated with BCL2-mediated cellular survival/ceramide-induced apoptosis, which corresponded with a favorable role for increased BCL2 expression in breast cancer treated with endocrine therapy. However, following data mining, the putative targets of these miRNAs did not demonstrate a specific mechanism and were not associated with the aforementioned pathways. Consequently, the authors proposed that the putative targets were not informative, and that further research was required to elucidate the exact mechanisms underlying the association between the identified miRNAs and the outcome of tamoxifen treatment (65).

Increasing levels of miRNA-26a were observed to be significantly (P<0.005) associated with clinical benefit and prolonged time to progression (TTP) in 235 patients with ER-positive tumors, who were administered tamoxifen as the first-line therapy for the treatment of metastatic disease (66). This indicated that miRNA-26a may serve as an optimal marker associated with the outcome of tamoxifen therapy. The authors performed an exploratory pathway analysis with a global testing approach (GTA) to identify cyclin and cell cycle regulation genes; cyclin E1 (CCNE1) and cyclin-dependent kinase 2 (CDC2) were the only genes that contributed significantly and overlapped between miRNA-26a and enhancer of zeste homolog 2 (EZH2). This study may aid clinicians in the identification of patients resistant to tamoxifen (66). This finding also provides rationale for the application of altered expression levels of specific miRNAs as a predictive marker for endocrine-resistant breast cancer.

However, another study using global miRNA analysis was performed on 152 ER-positive primary tumors from high-risk patients with breast cancer, who had received adjuvant tamoxifen as monotherapy and half of which had developed distant recurrence. Based on the large sample size, there was no single miRNA profile that was predictive of the outcome following adjuvant tamoxifen treatment in a broad cohort of ER-positive patients with breast cancer (41).

#### Limitations of the currently available studies

Regardless of the notable achievements in the previous studies concerned with the role of miRNAs in endocrine resistance (summarized in Table II), the implementation of miRNAs for clinical use remains at an early stage. Furthermore, the utilization of this information is limited by the following drawbacks of the available data.

The majority of the current findings are limited to cell line and animal models, although a small number of studies have been based on clinical specimens with a limited sample size. Moreover, the accuracy of the miRNA signatures has not been adequately evaluated, leading to difficulties in determining whether aberrant miRNA expression in breast cancer tissues is able to reliably differentiate endocrine-sensitive patients from endocrine-resistant patients. Additional studies are required to determine the roles of miRNAs in a clinical setting, to generate conclusive results. It is essential that the potential of the miRNA expression profiles is carefully validated prior to being adopted for clinical applications.

Furthermore, the differences in the expression levels of miRNAs have been observed in different endocrine-resistant cell lines, and should be further investigated to determine whether the differences are only associated with the characteristics of the cell types or caused by more complex mechanisms. Technical and analytical variations may contribute to inconsistencies between different miRNA profiling studies, and standardization and confirmation with further analytical approaches are critical for the validation of these findings.

In addition, the majority of the targets and signaling pathways in which miRNAs are involved have been predicted by computational approaches and require further investigation in functional studies. Mueller and Bosserhoff suggested that the lack of understanding with regard to the fundamental rules for the pairing of miRNAs to their target sequences leads to deficiencies in current miRNA target prediction algorithms. Identifying and verifying further miRNA-target gene interactions may improve the reliability of the present algorithms (67).

Moreover, the majority of studies have been based on microarray analysis, which detects significant expression through certain criteria. Potential miRNAs with essential functions that do not change significantly enough to meet the criteria may be ignored. Furthermore, it is unlikely that all differential expression patterns are equal. Among a number of up- or downregulated miRNAs, some are passengers and others are drivers, and this is a critical difference.

Additionally, it is beneficial to examine the sensitivity and specificity of miRNA expression profiles in clinical situations. Future studies should use adequate sample sizes to discover a large number of potential endocrine-resistant breast cancer-associated miRNAs. The specificity and sensitivity of any single miRNA may be limited; establishing a panel of symmetrical and validated miRNAs expression profiles may produce the best result.

Further, the main targets and regulators of miRNAs must be identified for the role of miRNAs in complex multistep endocrine resistance to be understood. As miRNA biogenesis is a multistage process, defects in miRNA processing have also been shown to enhance tumorigenesis (68). It may be hypothesized that the altered biogenesis of miRNAs is also a significant factor that contributes to the development of endocrine resistance in breast cancer. To the best of the authors’ knowledge, such research is not yet available. This is likely to provide an invaluable opportunity for studying the role of miRNAs in endocrine resistance.

## 6. Future prospects

Although miRNA research is in the early stage, there is rationale for speculating that miRNAs are likely to have a significant impact on improving the management of patients with breast cancer who exhibit endocrine resistance. With the accumulation of studies concerning the clinical significance of miRNAs, these small molecules are predicted to have potential for the development of novel predictive and therapeutic approaches.

### 

#### Prediction of the development of endocrine resistance

At present, endocrine resistance in breast cancer is predicted by utilizing the ‘wait and see’ approach. In this situation, endocrine resistance, which is measured radiographically, and appreciable changes in tumor recurrence and metastases may only be detected in the long-term, following the initiation of therapy, resulting in unacceptable and devastating outcomes. With miRNAs, responses and resistance to therapy are likely to be detected at an early stage during the course of treatment. As the technology evolves and is able to detect miRNAs with sufficient sensitivity and specificity, we suggest that it will allow the prediction of resistance to endocrine therapy.

Additional studies are required to determine whether the potential responses to specific endocrine treatment for patients with breast cancer may be efficiently classified by the patients’ miRNA expression profiles. If these are demonstrated to be sensitive enough to detect the early presence of endocrine resistance and absent or minimal resistance in endocrine-sensitive individuals in future clinical trials, miRNAs are likely to be promising biomarkers for predicting responses and resistance as patients with breast cancer receive endocrine therapy.

The advent of advanced technologies has allowed clinical tissues from patients of known responsiveness or resistance to endocrine therapy to be used as a means of obtaining an enhanced understanding of the potential mechanisms of endocrine resistance. miRNAs, which are stable molecules that have been shown to be well-preserved in formalin-fixed paraffin-embedded tissues, as well as fresh snap-frozen specimens (69), have enormous potential to serve as ideal biomarkers. However, the difficulty in obtaining the tumor tissue when the resistance has developed, as opposed to prior to therapy, retards this approach (24). Additionally, biopsies of patients with metastatic disease in the lung, bone or liver are complicated to perform, and are associated with high morbidity rates. However, such tissue is crucial for the molecular profiling of resistant tumors, in order to understand the underlying pathways.

miRNAs in circulation are readily accessible and may be sampled relatively noninvasively. The presence of miRNAs in serum was first described in patients with diffuse large B-cell lymphoma (70). Subsequently, several studies have identified similar results regarding the presence of miRNAs in circulation and their potential to serve as novel biomarkers for diseases and physiological states, including malignancy, diabetes mellitus and pregnancy (71–73). For breast cancer, the first example of genome-wide miRNA expression profiling in the circulation was by Zhao *et al,* who utilized microarray-based expression profiling to identify circulating miRNAs that were differentially expressed in 20 patients with breast cancer and 20 controls. The authors identified 26 miRNAs with at least two-fold differential expression between the cases and controls, indicating potential for the development of a signature of circulating miRNAs that may function as a diagnostic biomarker of breast cancer (74).

miRNAs in circulation enable rapid and repeated monitoring of miRNA expression profile changes through the course of endocrine treatments, which is likely to provide insights into the molecular evolution of the tumor. This, in turn, is likely to enhance the personalized treatment of endocrine therapy for patients with breast cancer.

#### Therapeutic potential

As the deregulation of miRNAs is involved in the development of endocrine resistance in breast cancer, there is potential for targeting miRNAs to serve as therapeutic guides for personalized medicine. miRNAs are promising in the field of drug development. in that they are likely to provide directions for overcoming endocrine resistance. With regard to the complex mechanism of endocrine resistance, the ability of miRNAs to regulate several target genes is particularly important. Thus, by targeting one miRNA, it is possible to target multiple pathways involved in the formation of endocrine resistance.

Strategies for regulating miRNA expression in endocrine resistant patients include the normalization of deregulated miRNAs and targeting specific miRNAs that may allow for the prevention or resensitization of resistance to hormonal therapies. Antisense oligonucleotides have been shown to block the function of miRNAs in numerous studies (75–77). Notably, the possibility of the delivery of antisense oligonucleotides *in vivo* was demonstrated by Krützfeldt *et al*, who showed that the intravenous administration of antisense oligonucleotides against miRNA-16, -122, -192 and -194 resulted in a significant reduction in the expression of the corresponding miRNA levels in various tissues (78). These results suggest that silencing specific overexpressed miRNAs involved in endocrine resistance *in vivo* may be a novel therapeutic strategy (available as antisense oligonucleotides against miRNAs, locked nucleic acids, anti-miRNAs or antagomirs). This process is theoretically similar for the downregulated miRNAs, and the restoration of these miRNAs may be another important therapeutic approach (available as miRNA mimics).

At present, clinical trials that utilize the miRNA approach to overcome endocrine resistance are not yet available (www.clinicaltrials.gov). However, before miRNAs become part of molecular therapies, further studies are required to determine their roles in patients, as the majority of the current findings are indirect, *in vitro* or both. With continuous research on the biological characteristics of miRNAs and the delivery systems, we propose that such strategies are likely to become available in the near future.

## 7. Conclusion

Differentially expressed miRNAs of endocrine-resistant patients with breast cancer may serve as potential biomarkers for endocrine-resistant tumors. Furthermore, the determination of the target genes/pathways of deregulated miRNAs further enhances the understanding of the mechanism of endocrine resistance and facilitates the development of novel targeted therapeutic agents. In addition to altered expression, the miRNAs themselves may be predictive of drug resistance development and utilized as biomarkers in the personalized clinical management of breast cancer.

Although significant progress has been made in clarifying the functional contribution of miRNAs to the modification of endocrine resistance in breast cancer, we are still in the infancy of our understanding. The limitations of the current studies are likely to be overcome by future continuous research via strategic selection and utilization of an already robust and expanding array of analytical tools. Further well-designed preclinical and clinical studies in this field are required. These scientific endeavors should lead to significant developments in the future management of endocrine resistance in patients with breast cancer. We propose that dynamic monitoring of circulating miRNAs through the course of endocrine therapy may facilitate the process.

## Figures and Tables

**Table I.** Potential targets of miRNA and pathway prediction tools.

Tool	Method for prediction and ranking	Website
Target Scan	Stringent seed pairing, conservation, UTR context	http://www.targetscan.org/
PicTar	Stringent seed pairing, free energy, conservation, probability being target to set of miRNAs	http://pictar.mdc-berlin.de/
miRanda	Moderately stringent seed pairing, free energy, conservation	http://www.microrna.org/
miR Base	Target predictions using miRanda algorithm with varied parameters	http://www.mirbase.org/
PITA	Seed pairing, site accessibility, total interaction energy, site number	http://genie.weizmann.ac.il/pubs/mir07/

UTR, untranslated region.

**Table II.** Summary of miRNAs involved in the endocrine resistance of breast cancer.

miRNAs	miRNA expression	Putative target	Drug	Sample source			Reference
				Cell line	Patient	Xenograft	
-221, -222	↑	p27Kip1	Tamoxifen	MCF-7	Yes	No	(9)
	↑	ERα	Tamoxifen	MCF-7/T47D MDA-MB-468	Yes	No	(48)
	↑	β-catenin, TGF-β	Fulvestrant	MCF-7	No	No	(53)
	↑	-	Tamoxifen	AK47	No	No	(50)
	↑	ERα, *PDCD4*, *BCL2*, *CYP1B1*, *ERBB3*	Tamoxifen	MCF-7	No	No	(49)
let-7i,-181a,-191-199b, -373^*^,-204, -211, -212, -216, -328,-424, -204, -191, let-7i	↓	TGF-β, Wnt MAPK, mTOR	Fulvestrant	MCF-7	No	No	(10)
-342	↓	cyclin B1, BRCA1 p53, PTEN	Tamoxifen	MCF-7	No	No	(55)
-222,-181b	↑	TIMP3, MAPK, AKT, ADAM10, ADAM17	Tamoxifen	MCF-7	Yes	Yes	(56)
-128a	↑	TGFβR1, SMAD2	Letrozole	MCF-7	No	No	(33)
-101	↑	Magi-2, Akt, PTEN	Tamoxifen	MCF-7	No	Yes	(61)
-210	↑	-	Tamoxifen	-	Yes	No	(64)
-15a/16	↓	BCL2	Tamoxifen	MCF-7	No	Yes	(58)
-30c	↑	HER, RAC1	Tamoxifen	-	Yes	No	(65)
-26a	↑	CCNE1, CDC2, EZH2	Tamoxifen	-	Yes	No	(66)
-451	↓	14-3-3 ζ, HER2, EGFR, MAPK	Tamoxifen	MCF-7	No	No	(60)

p27Kip1, cyclin-dependent kinase inhibitor 1B; ERα, estrogen receptor α; TGF-β, transforming growth factor β; PDCD4, pyruvate decarboxylase regulator; BCL2, B-cell CLL/lymphoma 2; CYP1B1, cytochrome P450 1B1; ERBB3, v-erb-b2 erythroblastic leukemia viral oncogene homolog 3; MAPK, mitogen-activated protein kinase; mTOR, mammalian target of rapamycin; BRCA1, breast cancer 1; PTEN, phosphatase and tensin homolog; TIMP3, tissue inhibitor of metalloproteinase 3; ADAM10/17, a disintegrin and metallopeptidase domain 10/17; TGFβR1, TGF-β receptor 1; Magi-2, membrane associated guanylate kinase; HER(2), human epidermal growth factor receptor 2; RAC1, ras-related C3 botulinum toxin substrate 1; CCNE1, cyclin E1; CDC2, cyclin-dependent kinase 2; EZH2, enhancer of zeste homolog 2; EGFR, epidermal growth factor receptor.
